# Role of Sulfonylurea Receptor 1 and Glibenclamide in Traumatic Brain Injury: A Review of the Evidence

**DOI:** 10.3390/ijms21020409

**Published:** 2020-01-09

**Authors:** Ruchira M. Jha, Josh Bell, Giuseppe Citerio, J. Claude Hemphill, W. Taylor Kimberly, Raj K. Narayan, Juan Sahuquillo, Kevin N. Sheth, J. Marc Simard

**Affiliations:** 1 Departments of Critical Care Medicine, Neurology, Neurological Surgery, Clinical and Translational Science Institute, University of Pittsburgh, Pittsburgh, PA 15201, USA; 2 Biogen, Cambridge, MA 02142, USA; josh.bell@biogen.com; 3 School of Medicine and Surgery, University of Milan-Bicocca, 20121 Milan, Italy; giuseppe.citerio@unimib.it; 4 Anaesthesia and Intensive Care, San Gerardo and Desio Hospitals, ASST-Monza, 20900 Monza, Italy; 5 Department of Neurology, University of California, San Francisco, CA 94110, USA; claude.hemphill@ucsf.edu; 6 Division of Neurocritical Care and Center for Genomic Medicine, Department of Neurology, Massachusetts General Hospital, Boston, MA 02108, USA; WTKIMBERLY@mgh.harvard.edu; 7 Department of Neurosurgery, North Shore University Hospital, Donald and Barbara Zucker School of Medicine at Hofstra/Northwell, Manhasset, NY 11030, USA; RNarayan@northwell.edu; 8 Neurotrauma and Neurosurgery Research Unit (UNINN), Vall d′Hebron Research Institute (VHIR), 08001 Barcelona, Spain; sahuquillo.juan@gmail.com; 9 Department of Neurosurgery, Universitat Autònoma de Barcelona (UAB), 08001 Barcelona, Spain; 10 Department of Neurosurgery, Vall d′Hebron University Hospital, 08001 Barcelona, Spain; 11 Division of Neurocritical Care and Emergency Neurology, Department of Neurology, Yale University School of Medicine, New Haven, CT 06501, USA; kevin.sheth@yale.edu; 12 Department of Neurosurgery, University of Maryland School of Medicine, Baltimore, MD 21201, USA

**Keywords:** SUR1 (Sulfonylurea receptor 1), TRPM4 (transient receptor potential melastatin 4), TBI (traumatic brain injury), cerebral edema, contusion expansion, glibenclamide, glyburide, ASTRAL

## Abstract

Cerebral edema and contusion expansion are major determinants of morbidity and mortality after TBI. Current treatment options are reactive, suboptimal and associated with significant side effects. First discovered in models of focal cerebral ischemia, there is increasing evidence that the sulfonylurea receptor 1 (SUR1)—Transient receptor potential melastatin 4 (TRPM4) channel plays a key role in these critical secondary injury processes after TBI. Targeted SUR1-TRPM4 channel inhibition with glibenclamide has been shown to reduce edema and progression of hemorrhage, particularly in preclinical models of contusional TBI. Results from small clinical trials evaluating glibenclamide in TBI have been encouraging. A Phase-2 study evaluating the safety and efficacy of intravenous glibenclamide (BIIB093) in brain contusion is actively enrolling subjects. In this comprehensive narrative review, we summarize the molecular basis of SUR1-TRPM4 related pathology and discuss TBI-specific expression patterns, biomarker potential, genetic variation, preclinical experiments, and clinical studies evaluating the utility of treatment with glibenclamide in this disease.

## 1. Introduction

The public health impact of traumatic brain injury (TBI) on death and disability is immense. With more than 50 million cases annually, TBI is projected to remain the leading global cause of neurological disability until 2030, surpassing Alzheimer’s disease and cerebrovascular disorders [1]. Although characteristics of the primary injury are generally considered non-modifiable, reducing the impact of secondary injury has the potential to substantially improve outcomes after TBI. Existing multivariable TBI clinical outcome prediction models from large patient populations such as IMPACT (International Mission for Prognosis and Clinical Trial design in TBI) explain ~35% of outcome variability [2,3]. These models predominantly utilize non-modifiable injury characteristics (demographics, severity, motor score, pupillary reactivity with some added value from admission computed tomography (CT), laboratory values and second insults). Thus, more than 50% of TBI outcome variability may be related to host-response and secondary injury processes, providing tremendous opportunity for targeted intervention.

Cerebral edema and contusion expansion are key secondary injury contributors to post-traumatic morbidity and mortality and for decades have commanded a significant proportion of neurosurgical and neurocritical care resources [4,5,6,7,8,9,10,11,12,13,14,15,16,17,18,19,20,21,22,23,24,25,26,27,28]. There is also evidence that traumatic microbleeds (previously considered markers of axonal injury) may represent vascular injury with perivascular iron laden macrophages tracking a network of injured vessels [29]—yet another permutation of vascular secondary injury that has unfavorable functional consequences and that may respond to targeted therapy [29,30,31,32]. Cerebral edema has been noted to occur in up to 60% of TBI patients with mass lesions and ~15% with initially ‘negative’ CT scans [13,33,34,35]. Contusion expansion, an extreme manifestation of vasogenic edema and blood brain barrier (BBB) breakdown, occurs in approximately 50% of cases [4,5,6,7,8,9]. Cerebral edema is a major cause of intracranial hypertension; these concepts are related via the Monro–Kellie doctrine, autoregulation, and pressure–volume relationships such as intracranial compliance and elastance [14]. While intracranial pressure (ICP) has been a focus of the Brain Trauma Foundation guidelines [16], it is an imperfect proxy for edema and treatment of the latter may become an important conceptual shift in the field as measures of cerebral edema (e.g., radiographic) improve. Nonetheless, depending on individual cerebral compliance, brain volume, and extent of edema development or lesion progression, both cerebral edema and contusion expansion can manifest as intracranial hypertension, irreversible brain injury, herniation, and death. Indeed, brain swelling, in large part due to cerebral edema and contusion volume, accounts for up to 50% of mortality in severe TBI [36,37,38].

Despite the extensive medical and financial burden, treatment options for cerebral edema and contusion expansion remain suboptimal, reactive, and largely unproven. There is no current treatment for diffuse axonal injury/traumatic microhemorrhages. Standard management protocols for cerebral edema and resultant intracranial hypertension after TBI frequently include hyperosmolar therapies (mannitol, hypertonic saline), sedation, hypothermia, neuromuscular paralysis, barbiturate coma, and decompressive craniectomy [14,16,39]. These therapies are neither targeted nor preventive, and they carry significant associated morbidities [14]. Some, such as decompressive craniectomy, are life-saving but do not stave off the underlying processes of secondary injury or functional impairment/disability [40,41,42]. Others, like hypothermia, may effectively control ICP but possibly increase the odds of worse outcome [43]. The modest mortality benefit proffered by tranexamic acid (TXA, 1.7% absolute risk reduction) in CRASH3 (a recent large randomized controlled trial), while promising, did not extend to severe TBI or result in an improvement in functional outcome [44]. Evidence is not yet available from this study regarding whether TXA mitigated hemorrhage progression; smaller studies have yielded conflicting data [45], and results pending publication from another large trial (NCT01990768, *n* = 987) disappointingly suggest no benefit on contusion expansion.

SUR1-TRPM4 is a unique cation channel emerging as a promising target for the treatment of cerebral edema and contusion expansion after TBI. In this narrative review, we briefly summarize molecular studies of SUR1-TRPM4, outlining the pathophysiology of this channel as it relates to cerebral edema and BBB integrity. We subsequently discuss SUR1-TRPM4 expression patterns in TBI and its potential utility as a theranostic biomarker. Research suggesting that genetic variation in *ABCC8* (encoding SUR1) and *TRPM4* may play a role in secondary injury and outcome after TBI is also presented. Finally, existing preclinical and clinical studies specific to SUR1-TRPM4 inhibition via glibenclamide (GLI) in TBI are reviewed.

## 2. Molecular Mechanisms of SUR1-TRPM4 in Models of CNS Injury

Over the last two decades, a growing body of preclinical and clinical research has demonstrated a crucial role of SUR1-TRPM4 in central nervous system (CNS) pathology, including cerebral edema, BBB integrity, and neuroinflammation.

### 2.1. SUR1-TRPM4, GLI, and Cerebral Edema

Sulfonylurea receptor 1 (SUR1), encoded by *ABCC8*, is a member of the adenosine triphosphate (ATP) binding cassette transporter superfamily that alone has no known function [46]. SUR1 itself is a regulatory subunit of multiple ion channels [47]. It undergoes obligate association with (and modulates) different pore-forming subunits to create heterologous ion channels in several cell types, of which SUR1-KIR6.2, an ATP sensitive potassium channel, has historically been the most extensively studied in the context of its activity in pancreatic β cells and role in diabetes mellitus. SUR1-KIR6.2 mediates potassium efflux and hyperpolarizes cells; channel blockade by GLI stimulates insulin release, thus leading to its utility in the treatment of diabetes mellitus [46,47,48,49]. Of note, SUR1-KIR6.2 is also expressed in the CNS, including after TBI [50,51].

**Figure 1:**
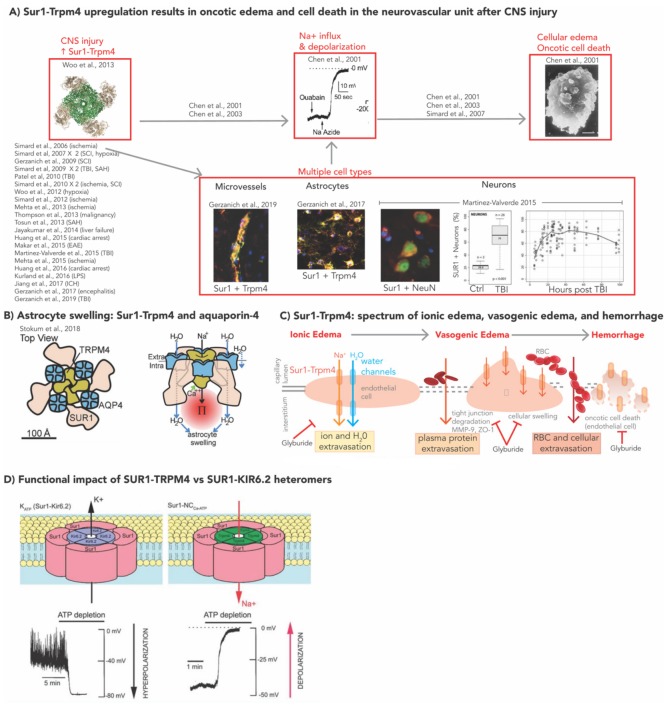
Overview of SUR1-TRPM4 and cerebral edema in central nervous system injury. (**A**) Sur1-Trpm4 upregulation results in oncotic edema and cell death in the neurovascular unit after CNS injury. This schematic contextualizes and lists the relevant research studies that have contributed towards understanding the basic SUR1-TRPM4 related molecular pathophysiology underlying cerebral edema and oncotic cell death after CNS injury. The processes themselves are highlighted in a red box: increased SUR1-TRPM4 expression in CNS injury has been reported in multiple cell types (microvessels, astrocytes, neurons). SUR1-TRPM4 activation results in sodium influx and cellular depolarization. This is associated with cellular edema, blebbing and oncotic cell death. Individual images adapted with permission from Chen et al., 2001, Martinez-Valverde et al., 2015, Gerzanich et al., 2017, Gerzanich et al., 2019 as referenced. (**B**) This is a proposed model of a SUR1-TRPM4-AQP4 hetero-multimeric complex showing AQP4 tetramers (blue channels) interposed within the SUR1-TRPM4 octamer (top view on the left, coronal view on the right). This model demonstrates SUR1-TRPM4 activation by increased intracellular Ca^2+^ (green arrow), which results in depolarization from Na^+^ influx and raised intracellular oncotic pressure. This, in turn, drives intracellular water influx (blue arrows) via AQP4 channels causing astrocyte swelling. Adapted with permission from Stokum et al., 2018. (**C**) Schematic showing the spectrum of ionic edema, vasogenic edema, and blood brain barrier destruction resulting in hemorrhage. The role of SUR1-TRPM4 is highlighted. Ionic edema, driven by osmotic forces, involves transcapillary flux of ions across the capillary membrane into the interstitium as demonstrated by luminal and abluminal SUR1-TRPM4 (orange) and water (blue) channels. Cytotoxic edema/cellular swelling (shown in the endothelial cell) involves movement of ions into cells creating an osmotic gradient that requires water to enter (in neurons, astrocytes, or endothelial cells). Vasogenic edema results from water and proteinaceous fluid traversing across a disrupted blood brain barrier that may occur in the setting of tight-junction degradation as well as oncotic edema of endothelial cells. In its extreme forms, when oncotic edema and cell death occurs in endothelial cells (shown) and astrocytes podocytes (not shown), complete breakdown of the blood–brain barrier results in erythrocyte extravasation, and hemorrhage into the interstitium. All these processes have been shown to involve SUR1-TRPM4, and thus may be inhibited by glibenclamide (also known as glyburide). (**D**)Schematic diagram of the two SUR1 regulated heteromeric channels expressed in the CNS after TBI: SUR1-KIR6.2 vs. SUR1-TRPM4. There are four subunits of SUR1 (pink) and four subunits of the pore-forming subunits KIR6.2 (lavender, left) or TRPM4 (green, right). ATP depletion activates both channels. SUR1-TRPM4 activation results in cell depolarization; the opposite effect (hyperpolarization) is achieved with SUR1-KIR6.2 activation. Adapted with permission from Simard et al., Sulfonylurea Receptor 1 in Central Nervous System Injury: A Focused Review. J Cereb Blood Flow Metab. 32 (9): 1699–1717. Copyright 2012 (SAGE Journals). CNS = central nervous system; EAE = experimental autoimmune encephalitis; ICH = intracranial hemorrhage; LPS = lipopolysaccharide; SAH = subarachnoid hemorrhage, SCI = spinal cord injury, TBI = traumatic brain injury.

In 2001, seminal patch-clamp experiments by Chen and Simard identified a novel SUR1-regulated non-selective cation channel in adult rat astrocytes [52,53]. In these experiments, channel opening was mediated by nanomolar concentrations of intracellular Ca^2+^ and ATP depletion (leading to its initial designation of NC_Ca-ATP_) [52,53]. This ion channel complex is now known to be a hetero-octameric structure comprising four SUR1 subunits, and four subunits of the pore-forming component, transient receptor potential melastatin 4 (TRPM4) [54]. SUR1-TRPM4 and SUR1-KIR6.2 have opposing physiological effects. SUR1-TRPM4 channel opening (e.g., by ATP depletion after treatment with sodium azide) is associated with indiscriminate influx of monovalent cations (predominantly sodium) and cell depolarization with subsequent cell swelling, blebbing, and oncotic cell death (Figure 1A) [46,52,53]. Recent work suggests that SUR1-TRPM4 co-assembles with the water channel, aquaporin-4 (AQP4), to form a hetero-multimeric complex that mediates water influx and resultant cellular swelling (Figure 1B) [55].

Unlike many other constitutively expressed SUR1 regulated channels in various systemic tissues, SUR1-TRPM4 is unique in that it is not normally present in the CNS, but undergoes de novo upregulation after CNS injury [36]. Upregulation of SUR1 with or without TRPM4 (hereafter SUR1±TRPM4) has been demonstrated in multiple CNS cell types (neurons, astrocytes, endothelial cells, macrophages, microglia) and models of injury, including ischemic stroke [56,57,58,59], TBI [50,60,61,62], spinal cord injury (SCI) [63,64], intracerebral hemorrhage (ICH) [65], subarachnoid hemorrhage (SAH) [66,67], CNS metastases [68], cardiac arrest [69,70], hepatic failure [71], and encephalomyelitis [72,73,74] (Figure 1A). The increased expression in several cell-types of the neurovascular unit renders SUR1-TRPM4 a likely contributor across the spectrum of cerebral edema endotypes (reviewed elsewhere [14,15,75]), including cellular/cytotoxic edema, ionic edema, vasogenic edema, and eventually hemorrhagic transformation/progression with complete disintegration of the BBB and oncotic death of endothelial cells (Figure 1C) [14,15,76].

Given its de novo upregulation, SUR1-TRPM4 inhibition has the potential to prevent or limit cerebral edema generation and hemorrhage progression at early stages of injury, with minimal side-effects. Channel blockade by gene silencing, deletion, or pharmacologic agents (primarily GLI) has demonstrated benefit in the aforementioned models of CNS injury [46,56,57,60,61,64,65,66,67,68,69,70,72,73,77,78,79,80,81,82,83,84,85,86,87,88]. GLI, also known as glyburide, inhibits SUR1-TRPM4 at nanomolar concentrations (EC_50_ = 48 nM) by increasing the probability of the channel’s long closed state (frequency and duration), not by affecting channel conductance [53]. This potency is increased eight-fold at acidic pH levels (~pH = 6.8) since GLI is a weak acid and protonation increases its lipid solubility [46,56,89]. Although GLI does not cross an intact BBB, the BBB is often disrupted in injured tissue (e.g., TBI, stroke). Moreover, the acidic pH of ischemic/injured CNS tissue facilitates drug transport across plasma membranes, and GLI can directly act on endothelial cells without traversing the BBB [46,56,89,90]. The effects of GLI on cerebral edema have been predominantly evaluated in ischemic stroke with promising results in edema reduction and BBB integrity in preclinical as well as clinical studies [56,57,78,89,91,92,93,94,95,96]. A Phase-3 trial of intravenous GLI (BIIB093) for malignant edema in ischemic stroke is ongoing (NCT02864953) [36]. Although less developed than the ischemic stroke literature, there is a rapidly growing body of research discussed in subsequent sections of this review, demarcating the importance of this channel and GLI in TBI. 

### 2.2. SUR1-TRPM4: Neuroinflammation and BBB Integrity

**Figure 2:**
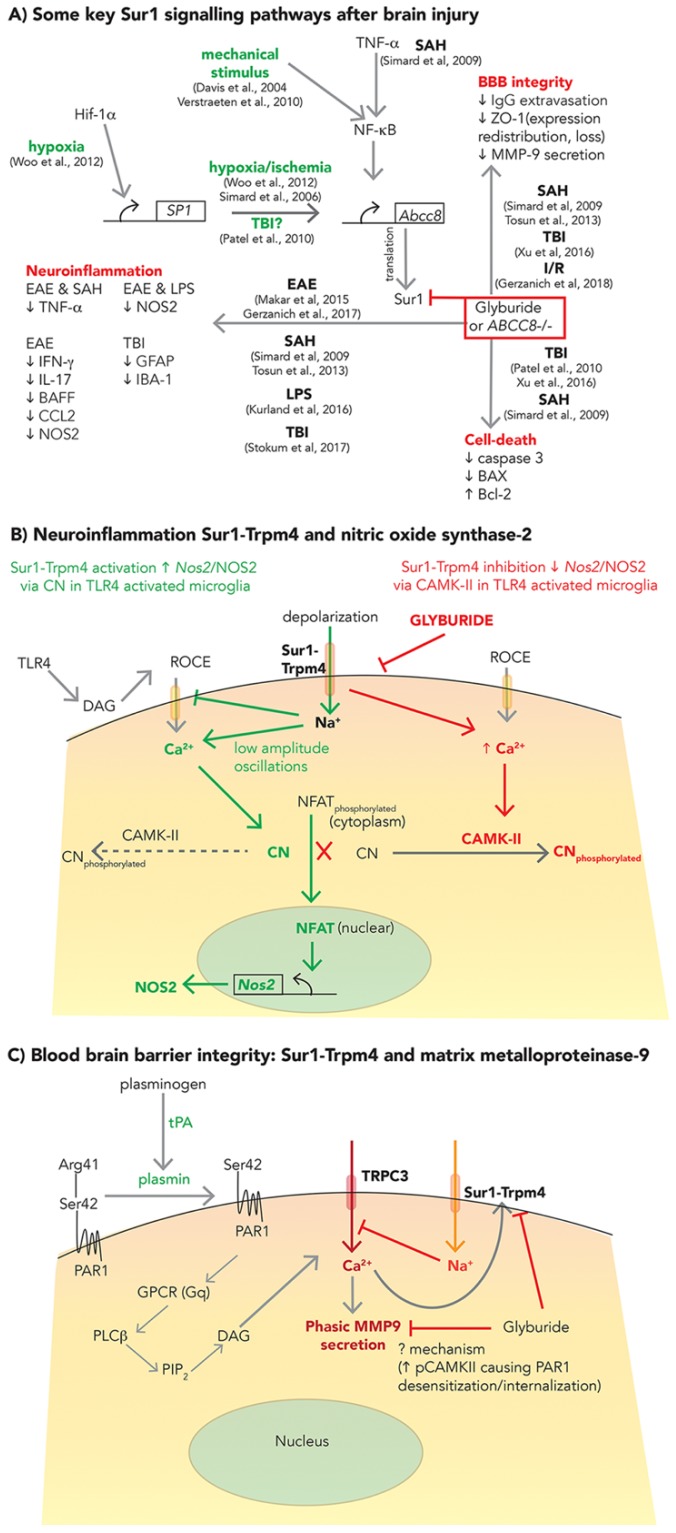
Putative SUR1 molecular signaling pathways beyond cerebral edema in brain injury. (**A**) Some key Sur1 signaling pathways after brain injury. This schematic illustrates upstream regulators of Sur1 (*Abcc8*) transcription such as Hif1-α, SP1 and TNF-α. The precipitating injury (e.g., hypoxia, TBI, mechanical stimulus) is listed in green. Downstream consequences of inhibiting upregulated Sur1 (via glyburide/glibenclamide or genetic knockout) on three major categories (blood brain barrier integrity, neuroinflammation, and cell death) are shown in red. Specific references that have demonstrated these effects are listed contextually and categorized by disease model. (**B**) Neuroinflammation, Sur1-Trpm4, and nitric oxide synthase-2 (NOS2). This illustration shows effects of Sur1-Trpm4 activation on nitric oxide (NO) production in microglial cells (pathway in green). Toll like receptor-4 (TLR4) activation induces de novo upregulation and activation of Sur1-Trpm4. Resultant depolarization, decreases the inward driving force for Ca^2+^, yielding low-amplitude repetitive oscillations of Ca^2+^. This activates calcineurin (CN), which dephosphorylates cytoplasmic NFAT and allows nuclear translocation. Nuclear NFAT is a transcription factor that upregulates transcription of Nos2, and thereby increases expression of NOS2 and production of NO. Inhibition of Sur1-Trpm4 by glyburide (glibenclamide) shown in the red pathway, increases intracellular calcium which activates calmodulin dependent protein kinase-II (CAMKII). CAMKII phosphorylates CN thereby inactivating it and preventing dephosphorylation and nuclear translocation of NFAT. Thus, Nos2 transcription is inhibited and NO production reduced. (**C**) Blood–brain barrier integrity: Sur1-Trpm4 and matrix metalloproteinase-9. Tissue plasminogen activator (tPA) converts plasminogen to plasmin, resulting in the cleavage (at Arg41) of protease activated receptor 1 (PAR1). Activated PAR1, via G protein receptor signaling (GPCR), phospholipase C (PLC), phosphatidylinositol biphosphate (PIP2) and diacylglycerol (DAG) activates transient receptor potential channel-3 (TRPC3, red) facilitating calcium entry and phasic MMP-9 secretion. Sur1-Trpm4 activation (orange) causing Na^+^ influx and depolarization, results in negative feedback of TRPC3, thereby inhibiting Ca^2+^ influx. Inhibition via glyburide (glibenclamide) despite increasing the drive for Ca^2+^ influx, reduces phasic MMP-9 secretion via a yet to be determined mechanism. One potential hypothesis includes phosphorylated CAMKII mediated PAR1 desensitization and internalization. BAX = Bcl-associated X protein; BCL2 = b cell lymphoma 2; CAMKII = calmodulin dependent protein kinase-II; CCL-2 = chemokine (C-C motif) ligand 2; CN = calcineurin; DAG = diacylglycerol; EAE = experimental autoimmune encephalitis; GFAP = glial fibrillary acidic protein; GPCR = G protein coupled receptor; HIF-1α = hypoxia inducible factor- alpha; IBA-1 = ionized calcium binding adaptor molecule 1; IFN-γ = interferon gamma; LPS = lipopolysaccharide; MMP-9 = matrix metalloproteinase-9; NFAT = nuclear factor of activated T-cells; NO = nitric oxide; NOS2 = nitric oxide synthase-2; PAR1 = protease activated receptor 1; PIP2 = phosphatidylinositol 4,5 biphosphate; PLC = phospholipase C; ROCE = receptor operated calcium entry; SAH = subarachnoid hemorrhage; SCI = spinal cord injury; Sp1 = specificity protein 1; SUR1 = Sulfonylurea receptor 1; TBI = traumatic brain injury; TLR4 = toll like receptor 4; TNF-α = tissue necrosis factor alpha; tPA = tissue plasminogen activator; TRPC3 = short transient receptor potential channel-3; TRPM4 = transient receptor potential melastatin 4; ZO-1 = zona occludens 1.

Some upstream molecular mechanisms related to SUR1-TRPM4 upregulation have been characterized in hypoxia/ischemia, SAH, and mechanical stress (Figure 2A) [67,97,98,99]. Beyond mechanisms of edema and oncotic cell death, SUR1-TRPM4 signaling and inhibition by GLI have also been identified as relevant in apoptotic cell death pathways involving Bcl-associated X protein (BAX) and caspase-3 (Figure 2A) [60,67,83], neuroinflammation (Figure 2A,B) [66,67,72,73,100,101], and BBB integrity (Figure 2A,C) via matrix metalloproteinase (MMP)-9 secretion, zona-occludens-1 (ZO-1) expression and redistribution [66,67,83,102] in different models of CNS injury, including SAH, TBI, ischemia-reperfusion (I/R), inflammation, and encephalomyelitis. However, the details are complex and remain incompletely understood, particularly in TBI. Nonetheless, they are important to explore given the potential implications for multifactorial benefits of SUR1-TRPM4 inhibition (e.g., with GLI) which may vary based on temporal, spatial and individual activation of these molecular networks.

#### 2.2.1. Neuroinflammation

The complex role of post-traumatic neuroinflammation involves both deleterious secondary injury as well as repair/recovery, thus making it a nuanced but important target for therapeutic modulation [103]. While not studied specifically in TBI, in vitro and in vivo models of lipopolysaccharide induced neuroinflammation demonstrate microglial activation of toll-like receptor 4 (TLR4), and de novo upregulation of SUR1-TRPM4, initiating a signaling cascade that increases nitric oxide (NO) production and contributes to harmful peroxynitrite-mediated protein radical formation [100,104,105]. TLR4 induced cellular depolarization via SUR1-TRPM4 decreases the inward driving force for intracellular Ca^2+^ influx. This results in low-amplitude repetitive oscillations in Ca^2+^, which activates calcineurin (a phosphatase). Calcineurin dephosphorylates nuclear factor of activated T-cells (NFATc1), allowing nuclear translocation and NFATc1-dependent upregulation of nitric oxide synthase-2 (Nos2) mRNA, NOS2 protein, and NO (Figure 2B, green) [100]. In this model, SUR1-TRPM4 inhibition (via GLI) increased intracellular Ca^2+^, as predicted, but reduced NFATc1, likely via persistent activation of calmodulin dependent protein kinase-II (CAMKII) (Figure 2B, red). Activated CAMKII phosphorylates and inactivates calcineurin, thereby preventing calcineurin dependent NFATc1 dephosphorylation and nuclear translocation. Here, GLI reduced microglial NOS2 expression/NO production, suggesting a mechanism by which it may be protective in other neuroinflammatory conditions such as TBI.

#### 2.2.2. BBB Integrity

Ongoing BBB breakdown perpetuates hemorrhage progression and contusion expansion after TBI. Although SUR1-TRPM4 contributes to BBB permeability via oncotic death of endothelial cells [14,15,76], it has also been implicated in this process in connection with other mechanisms involving ZO-1 [67,83], MMP-9 and/or tissue plasminogen activator (tPA) [91,95,102] (Figure 2A). MMP-9 is a zinc-dependent endopeptidase (collagenase) zymogen, widely implicated in extracellular matrix degradation and BBB disruption in many CNS diseases. However, its overlap with SUR1-TRPM4 requires further investigation, particularly in TBI [14,106]. While neutrophils are a major source of MMP-9 [107], it is also secreted from neurons, microglia, pericytes, and endothelial cells; indeed, it is prominently expressed in the microvascular endothelium during the first 24 h after focal ischemia [102,108]. In a model of I/R, tPA induced SUR1-TRPM4 channel opening and MMP-9 secretion from activated brain endothelial cells (Figure 2C) [102]. Conversion of plasminogen to plasmin (a serine protease) by tPA then induced canonical activation of protease activated receptor 1 (PAR1) by cleavage at Arg41. This triggered phasic MMP-9 secretion, and also opened SUR1-TRPM4 channels. The latter, via Na^+^ influx and a negative feedback loop, decreases the electrochemical driving force for intracellular Ca^2+^ influx. SUR1-TRPM4 inhibition by GLI (and shRNA against *Abcc8*) unexpectedly reduced phasic MMP-9 secretion from activated endothelium. Although the precise mechanism of the benefit remains unestablished, it is thought to be related to increased phosphorylated-CamKII and subsequent desensitization and internalization of PAR1 [102]. Nonetheless, it remains to be determined whether/to what extent this mechanism and GLI modulates BBB permeability after TBI. While other protease-mediated interactions (such as calpains) are also indisputably important contributors to BBB permeability after TBI, unlike MMP-9, they have not been evaluated in the context of SUR1-TRPM4 interactions. The link between caspases and SUR1-TRPM4 ± GLI have been evaluated in the context of apoptosis, not BBB permeability (Section 2.2).

## 3. SUR1-TRPM4 Expression in TBI

Patterns of increased SUR1±TRPM4 expression in post-traumatic human contusions are similar to those described in rat TBI models (Table 1) [50,51,60,61,62]. Scientific research understanding the complexity of SUR1’s association with its pore-forming subunit partners in TBI is limited, but rapidly evolving. As discussed in this section, previous studies have identified overexpression of KIR6.2 and SUR1 in post-traumatic human contusions (particularly astrocytes), however TRPM4 was not evaluated by this group [51,62]. In a separate study, TRPM4 overexpression was reported in a rodent model of TBI [109]. Determining precise temporal, spatial, and cellular expression patterns of both SUR1-TRPM4 and SUR1-KIR6.2 (and their functional implications if expressed simultaneously) after TBI is particularly important given the opposite effects of the two channels (Figure 1D). This will also impact their potential utility as biomarkers. In 2019, Gerzanich et al. evaluated SUR1, TRPM4, and KIR6.2 protein expression patterns in human contusion as well as rodent models of TBI; their findings are discussed in detail below (summarized in Table 1) [50]. Based on current information from these studies, SUR1-TRPM4 and SUR1-KIR6.2 multimers are not always expressed together after TBI, however simultaneous expression of SUR1, TRPM4, and KIR6.2 have been reported within certain cell-types [50].

In general, SUR1 colocalization (by immunofluorescence) and coassembly (by FRET imaging) with TRPM4 but not KIR6.2 has been noted in microvessels. Microglial cells and astrocyte tend to express all three channel subunits—SUR1, TRPM4, and KIR6.2—the functional implications of which are currently unestablished. Astrocyte expression has been most prominently noted in penumbral tissue. Increased endothelial expression of channel subunits typically preceded expression in microglia or astrocytes; this may impact the timing of edema endotypes (vasogenic±contusion expansion vs. cellular/cytotoxic), neuroinflammatory responses and repair mechanisms.

In the following subsections, expression of post-traumatic SUR1, TRPM4, and KIR6.2 is discussed—including ‘penumbral’ expression. A traditional understanding of traumatic ‘penumbra’ typically refers to brain tissue surrounding the primary lesion (e.g., contusion) that is damaged, ischemic, or susceptible to various forms of secondary injury but retains the potential for recovery i.e., the impact of insult on the ‘penumbra’ is not irreversible. However, this definition is likely to evolve and develop molecular sophistication. In early work on the SUR1-TRPM4 pathway, Patel et al., defined penumbra (*at time* = 0) as the region of tissue outside primary contusion where NFκB and SP1 were upregulated and secondary hemorrhage occurred. In this work, a parapenumbra was also identified where SP1 was upregulated but NFκB was absent—here, cell death may occur however hemorrhage progression was absent [60]. Recent work by the same group classifying human tissue (not at time = 0, but rather after secondary hemorrhage was completed), operationally defined the penumbra as GFAP+ and TUNEL− tissue [50]. Strictly speaking, since secondary hemorrhage had already occurred, this tissue would be more consistent with parapenumbra based on the definition used in the earlier study.

### 3.1. Increased SUR1±TRPM4 Expression in Rodent Models of TBI

To date, evaluation of SUR1±TRPM4 expression in preclinical TBI has occurred exclusively in rats and predominantly in models of focal cortical impact, with only a single study in diffuse central fluid percussion injury (FPI) [50,60,61,109]. Despite different TBI severity models in these studies, SUR1 [50,60,61] and TRPM4 [50,109] are consistently upregulated in different cell types of the neurovascular unit.

**Figure 3:**
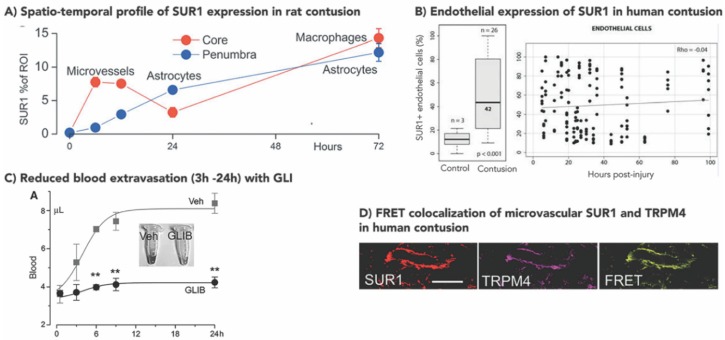
SUR1 expression and inhibition in contusion TBI. (**A**) Spatio-temporal profile of SUR1 expression in rat contusion. Graph of quantified SUR1 expression in core contusion (red) demonstrating prominent early expression in microvessels that decreases by 24 h, and increased expression in macrophages by 72 h. SUR1 expression in penumbra (blue) is predominantly astrocytic at all time points, with highest levels observed at 72 h. Adapted with permission from Gerzanich et al., 2019. (**B**) Endothelial expression of SUR1 in human contusion. The left panel shows a box-plot demonstrating a significantly higher proportion of SUR1+ endothelial cells in contusion vs. control samples (*p* < 0.001). The panel on the right demonstrates no correlation between % of SUR1+ endothelial cells and time since injury. Adapted with permission from Martinez-Valverde et al., 2015. (**C**) Reduced blood extravasation (3–24 h) with glibenclamide (GLI). Graph demonstrating mean quantified volume of extravasated blood at 0, 3, 6, 12, and 24 h (n = 3/group at all time points except 5/group at 24 h) in vehicle (grey squares) vs. GLI treated (black circles) rats after focal cortical impact. There is minimal increase in extravasated blood in the GLI treated group at 24 h vs. baseline. However, extravasated blood volume almost doubles by 24 h in vehicle rats. Differences between extravasated blood volumes in GLI treated vs. vehicle rats appear by 3 h, become significant by 6 h, and persist at 24 h. Error bars = standard error. ** = *p* < 0.01. Adapted with permission from Simard et al., 2009. (**D**) ImmunoFRET images from human contusion tissue for SUR1 (red) and TRPM4 (purple) demonstrating co-assembly of these heteromers (yellow) in a microvessel. Scale bar = 100 μm. Adapted with permission from Gerzanich et al., 2019.

In focal contusion (10 g weight, 5 cm drop, 1 m/s impact velocity), SUR1 expression was observed in tissue immediately beneath the impact as early as 3 h, predominantly in microvessels [61]. By 24 h, this became prominent in both neurons and capillaries in regions beyond the ipsilateral cortex, including the hippocampus, thalamus, and contralateral dorsomedial thalamus [61]. Further work in contusion by the same group (controlled cortical impact, CCI, 5 mm impactor, 4.5 mm depth, 1 m/s impact velocity) parsed out temporal and cellular differences in SUR1-TRPM4 expression in contusion core (GFAP–, TUNEL+) vs. penumbra (GFAP+, TUNEL–) as illustrated in Figure 3A [50]. In the core, SUR1 expression peaked at 6 h, remained elevated at 12 h, declined at 24 h and increased again at 72 h. During the first 24 h, SUR1 expression colocalized with TRPM4, and was predominantly noted in microvessels, which may have important implications for the treatment of vasogenic edema (see Section 3.5). There was no evidence of microvascular KIR6.2 expression. By 72 h, SUR1 expression was primarily noted in small round cells thought to be microglia/macrophages. Here, double labelling with TRPM4 or KIR6.2 demonstrated that these cells expressed all three channel subunits. In contrast, penumbral expression of SUR1 at all timepoints was noted in GFAP+ stellate cells thought to be astrocytes. These cells also expressed all three channel subunits: SUR1, TRPM4, and KIR6.2. Additional research is warranted to investigate the functional interactions and implications of this co-expression in both astrocytes and microglia/macrophages post-TBI, particularly since similar findings have been noted in human post-traumatic tissue (Section 3.2).

SUR1 protein and *Abcc8* mRNA expression in a milder model of focal impact were also elevated vs. sham [60]. Here, it was preceded by transcription factor specificity protein-1 (Sp1) expression, and was predominantly noted in hippocampal neurons at 6 h, peaking at 12 h, and declining by ~24 h post injury. While SUR1 expression has not been quantified in FPI, TRPM4 results were surprising in that increased expression persisted for up to 8 weeks after injury [109]. In this model, expression was noted in astrocytes, with differences noted between hippocampal grey vs. white matter (fissure). Higher TRPM4 levels were observed in the latter. TRPM4 expression was correlated with astrocyte swelling: TRPM4+ astrocytes had nearly double the soma size (*p* = 1.2 × 10^−12^) but did not undergo cell death. Future work exploring physiologic consequences of TRPM4 and/or KIR6.2 colocalization with SUR1 in this model could be valuable to guide therapeutic studies of channel inhibition in diffuse injury.

### 3.2. Increased Human SUR1±TRPM4 Expression in TBI

In a cohort of 26 patients with contusion, neuronal SUR1 expression was almost threefold greater in contusions vs. controls (meningioma, schwanomma, rhabdoid tumor, *p* < 0.001) [62]. While detected as early as 6 h post injury, neuronal SUR1 expression peaked at 24 h after which it stabilized. Early SUR1 expression was also detected in CD31 + endothelial cells (again, approximately threefold greater expression vs. controls). However, no temporal trend was noted in these cells; microvascular SUR1 expression remained elevated up to 100 h post-injury (Figure 3B). Semiquantitative immunohistochemistry did not detect any SUR1 expression in control GFAP+ glial cells (astrocytes), activated microglia/macrophages or neutrophils, whereas moderate SUR1 expression was found in all these cells-types after contusion. Most circulating neutrophils were SUR1 negative; however, those from brain parenchyma were SUR1 positive. The same group also identified KIR6.2 overexpression in contusional GFAP+ glial cells (astrocytes), consistent with findings in rodents. TRPM4 expression was not assessed in this study [51].

Deconstructing regional expression of SUR1, TRPM4, and KIR6.2 in contusion core vs. penumbra, analogous to the rodent experiments described above, yielded similar results [50]. Although temporal trends were not evaluated in human tissue, core lesions demonstrated SUR1 and TRPM4 expression, colocalization and heteromer coassembly (by FRET imaging) in microvessels (Figure 3D). KIR6.2 was not detected in these microvessels. SUR1 and TRPM4 colocalization was also demonstrated in CD68+ small round cells, i.e., microglia/macrophage; however, all three channel subunits (including KIR6.2) were expressed in these cells. Penumbral samples prominently expressed SUR1 in astrocytes, with minimal levels in microvessels or microglia/macrophages. TRPM4 and KIR6.2 levels were also increased in astrocytes, and FRET experiments demonstrated heteromers of both SUR1-TRPM4 and SUR1-KIR6.2. Neuronal expression was not evaluated in this study other than noting minimal expression of KIR6.2.

### 3.3. SUR1-TRPM4 Theranostic Biomarker Potential after TBI

**Figure 4:**
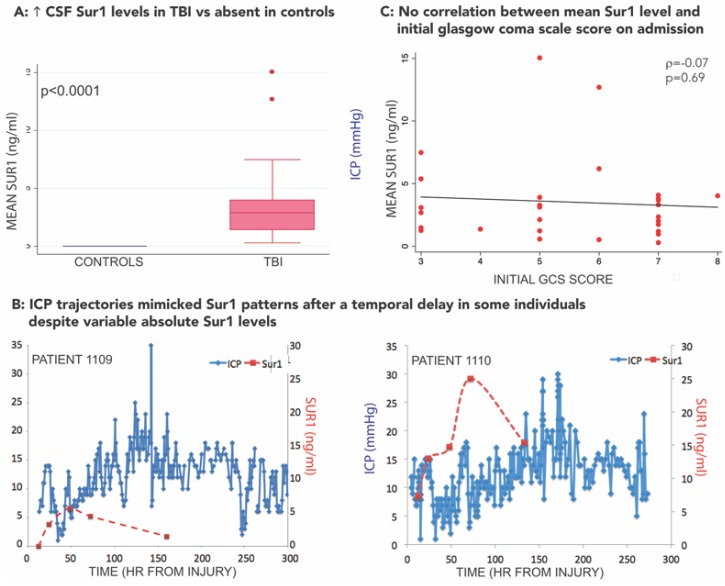
Cerebrospinal fluid SUR1 levels may be a biomarker for secondary injury after traumatic brain injury. (**A**) Box plot demonstrating increased cerebrospinal fluid (CSF) SUR1 levels (ng/mL) in TBI (red) vs. absent in controls. Error bars represent inter-quartile range. Adapted with permission from Jha et al., Sulfonylurea Receptor 1: A Novel Biomarker for Cerebral Edema in Severe Traumatic Brain Injury, Crit Care Med, 45 (3), e255–e264, 2017. (**B**) Temporal graph of SUR1 levels (ng/ml, red line) and intracranial pressure (ICP mmHg, blue line) in two patient examples (#1109, #1110). Here, the ICP trajectory mimics the SUR1 pattern after a variable temporal delay. Adapted with permission from Jha et al., Sulfonylurea Receptor 1: A Novel Biomarker for Cerebral Edema in Severe Traumatic Brain Injury, Crit Care Med, 45 (3), e255–e264, 2017. (**C**) Scatter plot demonstrating no correlation between mean Sur1 level (red dots) and initial Glasgow coma scale score (*x*-axis) on admission. Trendline shown in black. Pearson’s rho = 0.07, *p* = 0.69.

De novo upregulation of SUR1-TRPM4 after TBI (and other CNS disorders) affords it unique potential as a theranostic biomarker. However, serum ± CSF biomarker research regarding this pathway in TBI is currently in its infancy. Pilot work demonstrated markedly elevated SUR1 levels in cerebrospinal fluid (CSF) of 28 patients after TBI vs. undetectable levels in 15 controls with normal pressure hydrocephalus (Figure 4A) [110]. Remarkably, in some patients, ICP trajectories mirrored SUR1 expression patterns after a temporal delay (Figure 4B). SUR1 levels were associated with radiographic edema detected on CT scans, and with the initial degree of intracranial hypertension. SUR1 trajectories (rather than absolute values) were associated with both intracranial hypertension and outcome after TBI. No patients with declining SUR1 levels between 48–72 h had any episodes of intracranial hypertension or unfavorable outcome. Unfortunately, assessment of therapeutic intensity level was not available in this study, and the mechanism of SUR1 entry into the CSF remains unknown. While it is possible that the results relating SUR1 to measures of edema reflect an epiphenomenon, there did not appear to be a correlation between SUR1 level and initial injury severity as measured by the Glasgow coma scale (GCS) score (Figure 4C). Despite the multiple limitations of this exploratory study, including its single center and associative nature, the results are consistent with preclinical work and known pathophysiology in human studies, and warrant further exploration in larger cohorts. It is currently premature to consider SUR1 a biomarker in TBI, and further extensive functional and mechanistic studies are necessary to determine its utility. This work should include detailed phenotypic evaluation and take into consideration the multimeric expression of both SUR1-TRPM4 as well as SUR1-KIR6.2. Since SUR1 is the regulatory subunit of both these heteromeric channels (with opposite functional effects), the utility of SUR1 alone may be limited and dampen enthusiasm for clinical use in isolation. Additionally, it may be valuable to correlate SUR1-related biomarkers with several existing and promising biofluid markers of neuronal (ubiquitin C-terminal hydrolase-L1 [UCHL1], neuron specific enolase), astroglial (S100B protein, GFAP), axonal (neurofilaments, myelin-basic protein), and neurodegenerative (P- and T-tau) injury [111]. Since SUR1 heteromers with TRPM4 and KIR6.2 have been identified post-TBI in multiple cell types, correlation of biofluid levels with known markers of neuronal or astroglial injury may be informative. UCHL1 in particular may be valuable to correlate with SUR1-related biomarkers given its time course of upregulation over 24 h and its utility as an acute biomarker in severe TBI [111,112]. The discovery and validation of SUR1-pathway related biomarkers in the serum and CSF may become easier as the sensitivity and specificity of proteomic and transcriptomic technology improves. SUR1 quantification in serum/plasma samples after TBI has not yet been published, but represents another potentially important avenue for future research.

Other biomarkers related to SUR1-TRPM4 pathophysiology may also be important in TBI, but are currently understudied. In a recent Phase-2 trial of large hemispheric infarction, plasma MMP-9 levels were reduced in patients treated with intravenous GLI [91]. While largely unexplored in TBI, given the importance of MMP-9 in BBB integrity, vasogenic edema, and contusion expansion related secondary injury in this disease, MMP-9 may be a promising biomarker to investigate. A small study in 8 TBI patients demonstrated declining microdialysate MMP-9 levels over time, with a trend towards higher levels in patients with severe brain tissue hypoxia [113]. Another study in 12 TBI patients reported higher levels of pericontusional (vs. contralateral) microdialysate MMP-9 levels, but did not evaluate a relationship with cerebral edema or contusion expansion [114].

### 3.4. Impact of Genetic Variation in ABCC8 (Encoding SUR1) and TRPM4 in TBI

As mentioned earlier, approximately 65% of TBI outcome variability remains unexplained by existing large multivariable models that incorporate primarily non-modifiable injury characteristics identified on presentation [2,3]. It follows that the host-response is a key factor in regulating secondary injury processes such as cerebral edema and contusion expansion that significantly influence outcome after TBI. Genetic variation is increasingly recognized as an important contributor to variability in post-traumatic host response [28,115,116,117,118,119,120,121,122,123,124].

**Figure 5:**
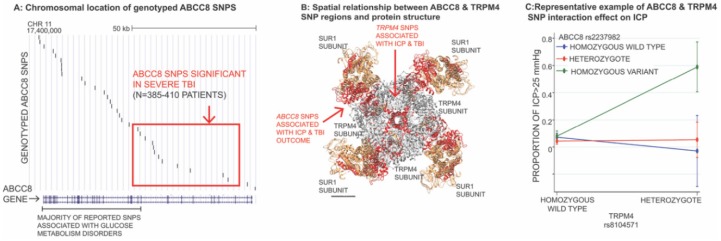
ABCC8 and TRPM4 genetic variation may impact edema, intracranial hypertension, and outcome post-TBI. (**A**) Chromosomal location of genotyped *ABCC8* single nucleotide polymorphisms (SNPs). Graphical display of the chromosomal location of sequenced (black lines) vs. significant (red box) SNPs as plotted on the UCSC genome browser (version GRCh38/hg38). The *ABCC8* gene is shown in blue, with vertical lines representing individual exons (and the interspersed horizontal lines representing introns) All the significant SNPs identified in TBI are upstream (introns 2–15) whereas the majority of SNPs significant in disorders of glucose metabolism (annotated in black) are downstream. Scale bar of 50 kb is shown. Adapted with permission from Jha et al., 2018. (**B**) Spatial relationship between *ABCC8* and *TRPM4* SNP regions and protein structure. This three-dimensional structure was created from UCSF Chimera automated software, combining the known crystallized structure of SUR1 (Li et al., obtained from the Research Collaboratory for Structural Bioinformatics Protein Data Bank [RCSB-PDB] using the ID 5WUA), with TRPM4 (Autzen et al., obtained from the RCSB-PDB using the ID 6BQV). In the putative octameric structure, the four SUR1 subunits are brown and the four TRPM4 subunits are gray. Amino acid sequences highlighted in red are those encoded by regions of *TRPM4* DNA (in proximity to rs8104571 and rs150391806), and those encoded by DNA regions in linkage disequilibrium with the eight significant *ABCC8* SNPs in TBI. (**C**) Representative example of *ABCC8* and *TRPM4* SNP interaction effect on ICP. This graph demonstrates the direction and magnitude of the interaction effect on ICP between *TRPM4* rs8104571 and *ABCC8* rs2237982. Here, patients homozygous wild-type for *TRPM4* rs8104571 have almost no proportion of their ICP measurements recorded at > 25 mmHg regardless of their *ABCC8* rs2237982 genotype. This is also true for patients heterozygous for *TRPM4* rs8104571 who are also wild-type for *ABCC8* rs2287982 (blue). Those who are heterozygous for *TRPM4* rs8104571 and are also heterozygous for *ABCC8* rs2287982 SNPs (red) have an average of ~5% of their ICP measurements recorded at > 25 mmHg. However, patients who are both heterozygous for *TRPM4* rs8104571 and homozygous-variant for *ABCC8* rs2237982, have an average of ~60% of their ICP measurements recorded at > 25 mmHg. Similar interaction effects between *TRPM4* rs8104571 and all three significant *ABCC8* SNPs in intron 10 (not shown) were obtained for multiple measures of ICP (including peak, average ICP values). Adapted with permission from Jha et al., 2018.

Targeted investigations into the impact of *ABCC8* and *TRPM4* genetic variation on cerebral edema, intracranial hypertension, and outcome after TBI have been performed in 385–485 patients with severe TBI from a single-center [28,122,123,124]. Genotyped *ABCC8* single nucleotide polymorphisms (SNPs) were distributed across the length of the gene with good coverage (Figure 5A) [122,123]. The majority of these were tag-SNPs (i.e., representative SNPs in high linkage disequilibrium [LD] with a group of SNPs/ haplotype). Eight regionally clustered *ABCC8* SNPs (rs4148622, rs1799857, rs11024286, rs2237982, rs2283261, rs2283258, rs3819521, rs7105832) were significantly associated with measures of CT edema, ICP and/or outcome (GOS) after TBI [122,123]. Minor allele frequencies were ~25–40%. Effect sizes were large and withstood the B-Y correction for multiple comparisons. These significant SNPs (and their associated 33 proxy-SNPs in LD) were all located upstream, between intron-2 and intron-15 of the 39 exon gene (Figure 5A) [123]. The spatially clustered polymorphisms were in LD with regions of DNA encoding the SUR1 site and transmembrane domain 0 (juxtaposing the TRPM4 binding site) (Figure 5B). This geography is in stark contrast to most SNPs reported in disorders of glucose metabolism, approximately two-thirds of which regionally cluster downstream towards introns/exons 16–39, in closer proximity with the adjacent gene *KCNJ11* (encoding KIR6.2). The true implications of the identified *ABCC8* SNPs in TBI remains unknown. Other than rs1799857 (synonymous variant in exon-12), the remaining significant SNPs are intronic, consistent with known SNP distributions in the human genome. Given their strategic spatial location, they may have important functional, splicing or gene expression/regulatory consequences. Further research is necessary to evaluate their impact in biological models and identify causal variants of disease.

Two *TRPM4* SNPs were associated with intracranial hypertension after TBI: rs8104571 and rs150391806. Effect sizes were large (e.g., 7–10 mmHg average ICP, 20–29 mmHg peak ICP with presence of the variant SNP), and they withstood corrections for multiple comparisons [124]. These were both rare variants with minor allele frequencies of 1% and 0.4%, respectively. Rs150391806 in exon 24 is a missense mutation. These two SNPs also demonstrated regional clustering (downstream) between intron 20 and exon 24 of the 25 exon *TRPM4* gene corresponding to regions of DNA that encode the TRPM4 channel pore and SUR1-TRPM4 binding interface (Figure 5B). However, as with *ABCC8*, the true functional impact of these variants is currently unknown. Of note, rs150391806 has also been associated with cardiac conduction disorders, including progressive familial heart block and Brugada syndrome [124].

A significant interaction effect has been reported between rs8104571 and three of the significant *ABCC8* SNPs, all located in intron-10 (rs2237982, rs2293261, rs11024286), that influences intracranial hypertension [124]. A representative example of this interaction is illustrated in Figure 5C, where patients heterozygous for *TRPM4* rs8104571 and homozygous-variant for *ABCC8* rs2237982 have ~60% of their ICP measurements >25 mmHg vs. ~0–5% in rs2237982 homozygous-wild types or heterozygotes. Those homozygous for *TRPM4* rs8104571 had almost no ICP recordings >25mmHg, regardless of their *ABCC8* rs2237982 genotype. This interaction effect was consistent across multiple measures of ICP where average ICP was ~30 mmHg vs. ~10 mmHg and peak ICP was ~60 mmHg vs. ~20–30 mmHg [124]. Virtually identical findings were reported for *TRPM4* rs8104571 interactions with all three of the intron-10 *ABCC8* SNPs across the different measures of ICP [124].

AQP4 genetic variation may also be relevant to cerebral edema pathophysiology related to SUR1-TRPM4. The role AQP4 in post-TBI edema generation is complex and multifaceted: AQP4 on perivascular foot processes is involved in increased intracellular water movement and cellular swelling, whereas decreased AQP4 expression on subpial, subependymal, and glymphatic surfaces reduces water elimination [14]. As mentioned earlier, SUR1-TRPM4 has been reported to co-assemble with AQP4 to form hetero-multimeric complexes mediating water influx and oncotic edema; this has not specifically been studied in models of brain contusion, but has been demonstrated in astrocytes after cold-injury to the cerebellum [55]. Interactions between genetic variations in all three protein structures may thus have major implications regarding edema formation. Previous candidate-gene research has demonstrated that polymorphisms in the AQP4 gene are related to outcome after TBI [116], but has not evaluated effects on measures of cerebral edema [125]. Nonetheless, AQP4 variants and mutations (including gain of function polymorphisms) have been reported to influence water permeability of cell membranes [126]. In ICH, AQP4 variant rs1054827 has been associated with perihematomal edema formation in a small sample of 128 patients [127].

Small sample sizes in TBI (particularly severe injury) have precluded unbiased genome wide association studies (GWAS) to identify novel genetic targets or validate the above findings. Despite 5 years and 11 enrolling sites, there are <300 patients with severe TBI in TRACK-TBI with genetic material available for evaluation. Efforts to increase sample sizes for genetic evaluation in TBI and validate these findings are paramount. These initiatives are currently ongoing in a large international multi-center collaboration known as GAIN (Genetic Associations in Neurotrauma) combining cohorts from TRACK-TBI (North America) and CENTER-TBI (Europe). Nonetheless, the reported findings are provocative, promising and consistent with known SUR1-TRPM4 pathophysiology. They support the theory that *ABCC8* and *TRPM4* genetic variation may play a role in cerebral edema development, intracranial hypertension and outcome after TBI. The spatial clustering of significant SNPs around critical regions of DNA encoding sequences contained in the channel pore, sulfonylurea receptor, and SUR1-TRPM4 binding interface, and the interaction effect between *ABCC8* and *TRPM4* variants moderating ICP adds further credence to this hypothesis. Validating effects of genetic variability in this pathway may facilitate risk stratification, prognostication, and guide patient selection for future trials.

### 3.5. Preclinical Studies of SUR1 Inhibition in TBI

A growing body of research in preclinical TBI has consistently demonstrated benefit of GLI on multiple post-traumatic outcome measures, including cerebral edema, BBB integrity, contusion expansion, and cognitive and motor function (Table 2) [50,60,61,82,83,84,85,101,128,129]. While GLI may improve motor and cognitive outcomes in models like FPI [128], and possibly cranial blast injury [101], the preponderance of preclinical evidence available to date suggests that its greatest value lies in treating contusions [50,60,61,82,83,85,129,130].

In different models, severities, and species of contusional TBI, GLI has improved BBB integrity and markedly reduced hemorrhage progression at multiple time points ranging from 45 min to 7 d [50,67,82,83]. In both mouse and rat CCI models (from two independent laboratories), extravasated blood from a leaky BBB at 24 h was approximately two fold greater in vehicle animals vs. those treated with GLI [61,83]. This difference with GLI treatment was notable by 3 h, became significant by 6 h post-injury, peaked at 12 h after which it stabilized by 24 h (Figure 3C) [61]. Reduced BBB leakiness was accompanied by reduced loss of ZO-1 and occludin in endothelial cells [83]. It should be noted that GLI-treated animals had minimal extravasated blood, and the amount quantified at 24 h was no different than that at 45 min, suggesting minimal progression of BBB breakdown with treatment [61]. Antisense oligodeoxynucleotide against SUR1 produced similar results in this study [61]. Not surprisingly, likely as a result of limited BBB breakdown, hemorrhagic progression of contusion by 24 h was reduced by ~58% [50]. Antisense oligodeoxynucleotides against *Abcc8*/SUR1 and *Trpm4*/TRPM4 but not *Kcjn11*/Kir6.2 reduced both hemorrhage progression and hemispheric swelling vs. controls after CCI [50]. Quantification of contusion volume over 7d of GLI treatment has demonstrated consistently lower volumes (~30–40%) at all time points (3 h, 24 h, 72 h, 7 d; all *p* < 0.01) [82]. GLI has also been tested by the rigorous, blinded, multi-center Operation Brain Trauma Therapy (OBTT) consortium, which evaluates promising therapies in three standardized preclinical models of TBI, including CCI [131]. Most drugs tested by OBTT performed “below or well below expectations based on the published literature” [132]. Although results from OBTT are pending publication for GLI, preliminary reports support a significant benefit in contusion volume reduction [85,130]. GLI has been identified by OBTT as the second highest scoring drug in the consortium (after levetiracetam), and the only agent tested by the consortium to reduce contusion/lesion volume in CCI [130].

GLI significantly decreases cerebral edema 24 h after CCI [50,82,83]. In two of the three studies, there was a 52–66% reduction in ipsilateral hemispheric swelling [50,83]. GLI also reduces diffuse (contralateral) edema exacerbated by detrimental second insults such as hypotension [84]. Most studies evaluating effects of GLI on post-traumatic cerebral edema have used percent brain water (%BW) for quantification. While this is considered the gold standard measure, it is biased against detection of cellular edema. In order to detect increases in %BW, some degree of vasogenic edema/BBB breakdown and perfusion is required; isolated cellular swelling in the absence of this is merely redistribution of water from the interstitial to intracellular space without actually increasing total water content [15]. MRI endophenotyping of edema may be valuable to evaluate such granular effects of SUR1-TRPM4 blockade. Pilot data from a combined injury mouse model of CCI plus hypotension indicate reduction of T2 hyperintensity and volume (measuring vasogenic edema) after treatment with GLI [129]. This was noted as early as 3 h post injury in some mice, and statistically significant at 24 h. GLI effects on cellular swelling measured by mean diffusivity are currently unknown in preclinical models, but MRI based outcome measures may facilitate quantification of cytotoxic edema. Thus, although results of GLI on cerebral edema as measured by %BW are promising, this method may underestimate potential benefits of SUR1-TRPM4 blockade on cellular swelling, e.g., in astrocytes. In a model of FPI, TRPM4+ astrocytes had double the soma size of TRPM4– astrocytes after injury, as assessed by confocal and electron microscopy. While an important methodology for evaluating preclinical benefit on cellular edema and proof-of-concept, microscopy-based techniques are less clinically translatable than radiographic metrics.

Upregulation of SUR1-TRPM4 in endothelial cells as early as 3 h (peaking before 24 h) coincides with the timing of the GLI benefit on vasogenic edema, BBB breakdown, and contusion expansion after preclinical contusional TBI. Based on this pathophysiology, early treatment with GLI is likely imperative to maximize therapeutic benefit on this process. Given delayed SUR1-TRPM4 upregulation in astrocytes and microglia/macrophages relative to endothelial cells, it is possible that continued treatment with GLI may influence late edema formation and/or neuroinflammation and repair. However, this hypothesis requires further investigation, particularly given the unknown implications of concurrent SUR1-KIR6.2 expression in some cell-types.

### 3.6. Clinical Studies of GLI in TBI

Three small clinical trials evaluating GLI in TBI have been published (Table 3). Two of these studies, conducted in Iran, explored effects of oral GLI in different subtypes of TBI, including contusion-TBI and diffuse axonal injury(DAI)/traumatic microhemorrhages.

Zafardoost et al., randomized 40 patients with moderate-severe DAI to 1.25 mg oral GLI every 12 h (this dose was increased to 2.5 mg if no hypoglycemia was noted at the lower dose) vs. control (no placebo) [133]. Power calculations were designed to detect an effect size of Cohen’s d ≥ 0.7 with α = 0.05 and 1 − β = 0.80 in this sample size. The distribution of presenting GCS scores between treatment vs. control groups is not clearly presented (although reported to not be significantly different). Outcome was assessed at 1 week and at discharge using ordinal measures of GCS and the Glasgow outcome scale (GOS) score, all of which were significantly better in the treated group vs. control (all *p* ≤ 0.004). Long term functional outcomes were not evaluated.

A blinded placebo-controlled randomized trial of oral GLI in contusion-TBI was performed by Khalili et al. in 66 patients with moderate-severe TBI (GCS 5–12) [134]. Multiple outcome measures were obtained, including contusion volumes (initial, day-3, day-7), and 3-month ordinal functional outcome metrics, including GOS, modified Rankin Scale, and disability rating scale. To have 80% power at an α = 0.05, a sample size of 60 was required to detect a 5% difference in outcomes. Patients were randomized to placebo or 10 mg oral GLI for 10 days. No differences were noted in any of the functional outcome measures or absolute contusion volumes. Patients treated with GLI had significantly lower contusion expansion ratios between both baseline-day3 (*p* < 0.001), and baseline-day 7 (*p* < 0.003).

Although pharmacokinetic values were not reported in the studies by Zafardoost et al., or Khalili et al., intermittent oral GLI dosing can be problematic, with sharp supratherapeutic peaks (risking hypoglycemia) and prolonged subtherapeutic troughs [36]. Variability in stomach pH can further influence plasma levels of the drug [36,94]. A small Phase-2 multi-center randomized trial reported by Eisenberg et al., utilized an intravenous formulation of GLI, currently known as BIIB093 [135]. This drug has also been used in a published Phase-2 trial in large hemispheric infarction (LHI) and is being evaluated in an ongoing Phase-3 trial of LHI (NCT02864953) as well as a large Phase-2 contusion-TBI trial (NCT03954041) [91,135]. The intravenous formulation enables rapid and stable plasma levels, reducing the risk of hypoglycemia and maximizing therapeutic duration.

The radiographic findings by Eisenberg et al. (with BIIB093) are similar to those reported by Khalili et al., in contusion [135]. Here, 28 patients (GCS 4–14) were randomized within 10 h of TBI to a 72 h infusion of placebo or BIIB093. Of these, only 14 patients had contusional TBI. Primary outcome was MRI-defined edema and or hemorrhage on a 1.5T scanner at 72 ± 12 h. GOS-extended at 90 days was a pre-specified secondary outcome. Analysis of contusions (7/group) revealed a 1036% increase in lesion volume (hemorrhage plus edema) in placebo vs. 136% in controls; this tenfold difference was not significant (*p* = 0.15), likely due to small sample size and contusion heterogeneity. It should also be noted that baseline contusion volumes were much higher in the treatment group of this study. Hemorrhage volumes decreased 29.6% with BIIB093 vs. an 11.6% increase with placebo; however, this was not significant. Nonetheless, one may speculate that the pathophysiologic basis for this relates to GLI enhanced RBC phagocytosis by macrophages (Simard et al., [136]). MRI measures of edema in this study included mean diffusivity (MD) as a proxy for overall water diffusivity, free water (FW) as a proxy for extracellular water, and tissue MD (MDt) as a proxy for intracellular water. These measures were compared in lesions vs. uninjured white matter (WM). Untreated lesions had increased edema by all three measures vs. uninjured WM (*p* < 0.02). In BIIB093 treated patients, there was no difference in any of these measures vs. uninjured WM. There was no improvement in 90-day functional outcome with BIIB093 vs. placebo in this small cohort; however, the study was not powered for this metric.

**Figure 6:**
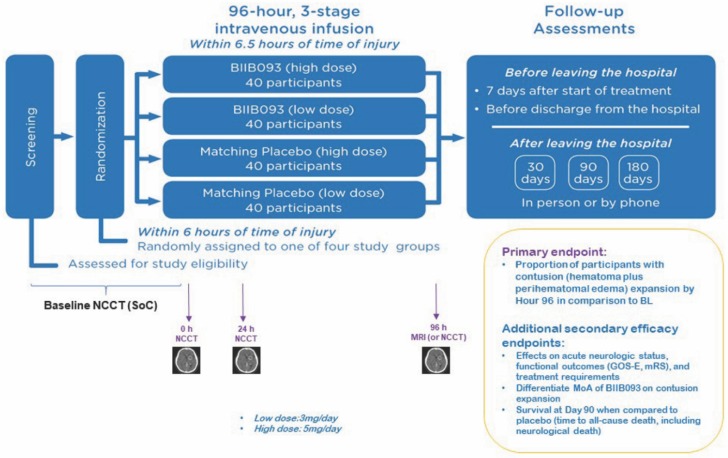
ASTRAL clinical trial design schematic. Diagram outlining the clinical design trial for ASTRAL, a Phase-2 multicenter, international, double-blind, multidose, placebo-controlled, randomized clinical trial evaluating the safety and efficacy of BIIB093 in supratentorial contusion-TBI (NCT03954041, Antagonizing **S**UR1-**T**RPM4 to reduce the progression of intracerebral hematoma and edema surrounding lesions). Distribution of participants to high vs. low dose BIIB093 vs. matching placebo is presented; patients are randomized within 6 h of time of injury. The primary end point is documented by 96 h MRI or non-contrast CT and related to proportion of participants with contusion expansion vs. baseline. Additional secondary efficacy endpoints will also be obtained as illustrated. Time-points for key follow up assessments are also shown including 7 days after treatment, prior to hospital discharge, 30-day, 90-day, or 180-day evaluations.

The compelling molecular and preclinical evidence supporting the benefit of GLI, particularly in contusion models, combined with encouraging clinical data and radiographic results in this subcohort despite very small studies, has generated excitement to test this therapy in a larger Phase-2 study of contusion-TBI. ASTRAL is a multicenter, international, double-blind, multidose, placebo-controlled, randomized Phase-2 clinical trial evaluating the safety and efficacy of BIIB093 in supratentorial contusion-TBI (NCT03954041, antagonizing SUR1-TRPM4 to reduce the progression of intracerebral hematoma and edema surrounding lesions). The trial is sponsored by Biogen and is actively enrolling patients. The proposed enrollment is 160 patients across several centers in North America, Europe, and Japan. Two doses will be tested (3 mg/day and 5 mg/day × 96 h) with matched placebo groups for each dose. In addition to extremely focused patient selection (contusion-TBI), the ASTRAL trial design leverages the mounting evidence that GLI reduces cerebral edema and hemorrhage progression, and has identified its primary outcome to be contusion expansion by 96 h (Figure 6). Multiple secondary outcomes will also be obtained, including acute neurological status, functional outcome, and survival. The temporal course of microvascular SUR1-TRPM4 expression and known risk-window for contusion expansion has also informed trial strategy: the treatment window for BIIB093 is thus 6.5 h. This is significantly earlier than 10 h in the aforementioned BIIB093 trial in TBI reported by Eisenberg et al. [135], as well as the aforementioned ongoing Phase 3 study of BIIB093 in LHI (NCT02864953).

## 4. Conclusions

There is substantial preclinical and clinical evidence supporting a pivotal role of the SUR1-TRPM4 pathway in cerebral edema development, contusion expansion, and outcome after TBI. Consistent findings of benefit with GLI treatment—particularly in contusion across models, injury severity, species, and drug doses—suggests robustness and is encouraging for successful clinical translation. Results from ASTRAL, the ongoing Phase-2 trial of intravenous GLI (BIIB093) in brain contusion are eagerly awaited. Future research on theragnostic biomarkers and identifying/validating functionally consequential genetic variants in this pathway may eventually inform clinical risk-stratification, prognostication, identification and characterization of treatment-responders, patient selection for precision-medicine based clinical trials, and the development of novel gene-based therapies.

## Figures and Tables

**Table 1:** SUR1, TRPM4, and KIR6.2 expression in rodent and human contusional TBI.

	Rat Contusion			Human Contusion		
	Increased (Y/N)	Peak (h Post Injury)	Ref	Increased (Y/N)	Peak (h Post Injury)	Ref
**SUR1**						
Neurons	Y	12–24 h	Simard et al., 2009 [61] Patel et al., 2010 [60]	Y	24 h	Martinez-Valverde et al., 2015 [62]
GFAP+ Glia (Astrocytes)	Y	72 h (penumbra)	Gerzanich et al., 2019 [50]	Y	↑ with time (100 h) GFAP+ penumbra (prominent)	Martinez-Valverde et al., 2015 [62] Gerzanich et al., 2019 [50]
Microvessels	Y	6–12 h (core)	Gerzanich et al., 2019 [50]	Y	stably ↑ GFAP- core	Martinez-Valverde et al., 2015 [62] Gerzanich et al., 2019 [50]
Microglia	Y	72 h (core)	Gerzanich et al., 2019 [50]	Y	↑ with time (100 h) GFAP- core	Martinez-Valverde et al., 2015 [62] Gerzanich et al., 2019 [50]
**TRPM4**						
Neurons	Unk	-	-	Unk	-	-
GFAP+ Glia (Astrocytes)	Y	72 h (penumbra)	Gerzanich et al., 2019 [50]	Y	GFAP+ penumbra	Gerzanich et al., 2019 [50]
Microvessels	Y	24 h	Gerzanich et al., 2019 [50]	Y	GFAP- core	Gerzanich et al., 2019 [50]
Microglia	Y	72 h (core)	Gerzanich et al., 2019 [50]	Y	GFAP-core GFAP+ penumbra (some)	Gerzanich et al., 2019 [50]
**KIR6.2**						
Neurons	Unk	-		N	-	Castro et al., 2018 [51] Gerzanich et al., 2019 [50]
GFAP+ Glia (Astrocytes)	Y	72 h (penumbra)	Gerzanich et al., 2019 [50]	Y	Unk GFAP+ penumbra	Castro et al., 2018 [51] Gerzanich et al., 2019 [50]
Microvessels	N	-	Gerzanich et al., 2019 [50]	N	-	Castro et al., 2018 [51] Gerzanich et al., 2019 [50]
Microglia	Y	72 h (core)	Gerzanich et al., 2019 [50]	Mixed Y	- (not different vs. controls) GFAP- core	Castro et al., 2018 [51] Gerzanich et al., 2019 [50]

h = hour, N = no; unk = unknown; Y = yes, ↑ = increased.

**Table 2:** Preclinical Studies of Glibenclamide in TBI (Chronologically Listed).

Authors, Year	Study Title	TBI Model	GLI Dose	Results
Simard et al, 2009 [61]	Key Role of Sulfonylurea Receptor 1 in Progressive Secondary Hemorrhage after Brain Contusion	Rat Focal Cortical Contusion 4.5 mm impactor 10 g weight drop 5 cm distance 1 m/s impact velocity	Loading: 10 μg/kg IP Maintenance: 200 ng/h	SUR1 upregulation 3 h (capillaries), 24 h (neurons, capillaries) Vehicle blood extravasation (contusion expansion, double by 12 h) > GLI group (minimal expansion) at 6 h, 12 h, 24 h ↓ 24 h histological lesion volume in GLI vs. vehicle Improved outcome (spontaneous vertical exploration) with GLI
Patel et al., 2010 [60]	GLI Reduces Hippocampal Injury and Preserves Rapid Spatial Learning in a Model of TBI	Rat Cortical Impact Injury Modified Feeney Device 10 g weight drop 3 cm distance 0.77 m/s impact velocity	Loading: 10 μg/kg IP Maintenance: 200 ng/h X 7d	Hippocampal SUR1 upregulation 6 h, peak 12 h ↓ hippocampal caspase-3 labeling with GLI Improved memory retention (hidden platform trial) with GLI treatment 3X reduction in FJC+ hilar cell death with GLI treatment
Zweckberger et al., 2014 [82]	GLI Reduces Secondary Brain Damage After Experimental TBI	Rat CCI 5 mm impactor 1.5 mm depth 7.5 m/s impact velocity 300 ms impact duration	Loading: 10 μg/kg IP Maintenance: 200 ng/h	GLI did not improve intracranial pressure, cerebral microdialysis parameters, seizures, or motor function (beam walk) GLI reduced 24 h brain water content (ipsilateral) by 15.3% GLI reduced contusion volume at 8, 24, 72 h and 7 d
Xu et al., 2016 [83]	GLI Attenuates Blood Brain Barrier Disruption in Adult Mice after TBI	Mouse CCI 3 mm impactor 1.5 mm depth 1.5 m/s impact velocity	10 μg/day X 3 days	GLI reduced 72 h ipsilateral brain water by 51.6% GLI reduced BBB disruption GLI inhibited loss of ZO-1 and reduced early stage apoptosis induced by stretch injury
Stokum et al., 2017 [101]	GLI Pretreatment Protects Against Chronic Memory Dysfunction and Glial Activation in Rat Cranial Blast TBI	Rat Direct Cranial Blast TBI 0.22 caliber cartridge Peak pressure ~427kPA 25.4 mm blast wave	Loading: 10 μg/kg IP Maintenance: 200 ng/h X 7d	GLI did not improve transient neurobehavioral/vestibulomotor deficits GLI pretreatment improved hippocampal memory function GLI pretreatment improved chronic hippocampal neuroinflammation
Jha et al., 2018 [84]	GLI Produces Region Dependent Effects on Cerebral Edema in a Combined Injury Model of TBI and Hemorrhagic Shock in Mice	Mouse CCI 1 mm depth 5 m/s impact velocity ± Hemorrhagic shock (HS) MAP 25–27 mmHg X 20 min	Loading: 20 μg/kg IP Maintenance: 400 ng/h	GLI did not improve 24 h ipsilateral brain water in CCI or CCI+HS GLI reduced contralateral brain water to sham levels after CCI+HS (no contralateral edema developed in CCI alone) GLI had no effect on 72 h ipsilateral brain water in CCI+HS, contralateral edema had resolved by this time-point.
Gerzanich et al., 2019 [50]	SUR1, TRPM4 and KIR6.2 Role in Hemorrhagic Progression of Contusion	Rat CCI 5 mm impactor 4.5 mm depth 1 m/s impact velocity 200 ms impact duration	Loading: 10 μg/kg IP Maintenance: 400 ng/h	↓ GLI hemorrhagic progression of contusion by 58% ↓ GLI mean swelling by ~66%
Bramlett et al [128] Deng-Bryant et al Jha et al., (abstracts 2015), [129] Jha et al (in preparation)	Operation Brain Trauma Therapy: GLI Treatment in TBI	Rat CCI Rat FPI I, 1.8–2.1 atm) Rat PBBI	Loading: 10 μg/kg IP Maintenance: 200 ng/h X 7d	GLI improved motor function (beam balance, walk) after CCI GLI improved motor function (cylinder task) after FPI GLI reduced 21d contusion volume after CCI GLI did not improve motor, cognitive, or histopathologic outcome after PBBI

BBB = blood brain barrier; CCI = controlled cortical impact, FJC = Fluoro Jade-C; FPI = fluid percussion injury; GLI = glibenclamide; PBBI = penetrating ballistic-like brain injury; SUR1 = sulfonylurea receptor 1; TBI = traumatic brain injury; ZO-1 = zona occludens-1; ↓ = decrease.

**Table 3:** Clinical Studies of Glibenclamide in TBI (Chronologically Listed).

Authors, Year	Study Title	Study Design	Sample Size	Outcome
Zafardoost et al., 2016 [133]	Evaluation of the Effect of Glibenclamide in Patients with DAI Due to Moderate to Severe Head Trauma	Randomized Controlled Trial DAI, GCS ≤ 12 Dose: 1.25 mg oral GLI Q12H (2.5 mg Q12H if no hypoglycemia) Duration: 7 days or ICU discharge	N = 40	GLI was associated with better GCS score 1 week post-trauma (*p* = 0.003) and at discharge (*p* = 0.004) GLI was associated with a better GOS score at discharge (*p* = 0.001) GLI was associated with shorter hospital length of stay (*p* = 0.03)
Khalili et al., 2017 [134]	Effects of Oral Glibenclamide on Brain Contusion Volume and Functional Outcome of Patients with Moderate and Severe TBI: A Randomized Double Blind Placebo Controlled Clinical Trial	Double-Blind Randomized Controlled Trial Moderate-Severe TBI (GCS 5-12) Dose: 10 mg/day oral GLI Duration: 10 days	N = 66	No difference in contusion volume between GLI vs vehicle at baseline, day 3 and day 7 GLI reduced contusion volume expansion ratio between day 1 vs. 3 (*p* < 0.001) and day 1 vs. 7 (*p* = 0.003) GLI did not improve functional outcome at 3 months as measured by GOS, MRS or DRS
Eisenberg et al., 2019 [135]	Magnetic Resonance Imaging Pilot Study of Intravenous Glyburide in TBI	Phase-2 Randomized Controlled Trial Dose: IV 0.13 mg load over 2 min, 0.16 mg/h for 6 h, 0.11 mg/h for 66 h Duration: 3 days	N = 28 (N = 14 contusion)	Contusion growth was non-significantly increased (10 fold) in placebo (1036 ± 1963.28%) vs. GLI (136 ± 195.62%; *p* = 0.15, n = 7/group) MRI measures of edema (FW, MD, MDt) in lesion vs. uninjured WM were different in placebo (*p* < 0.02) but not GLI
NCT03954041	Antagonizing SUR1-TRPM4 To Reduce the progression of intracerebral hematoma And edema surrounding Lesions (ASTRAL)	Multicenter Double-Blind Multidose Placebo-Controlled Randomized Trial (Phase-2) Drug Administration: IV bolus+ infusion Low Dose Arm: 3 mg/day High Dose Arm: 5 mg/day Duration: 96 h	Estimated N = 160	Currently enrolling

DAI = Diffuse Axonal Injury; DRS = Disability Rating Scale; FW= free water; GCS = Glasgow Coma Scale; GLI = glibenclamide; GOS = Glasgow Outcome Scale; IV = intravenous; MD = mean diffusivity; MDt = tissue mean diffusivity; MRI = magnetic resonance imaging; MRS = Modified Rankin Scale; WM = white matter.

## Abbreviations

AQP4	aquaporin 4
ATP	adenosine triphosphate
BAX	Bcl-associated X protein
BBB	blood brain barrier
CAMKII	calmodulin dependent protein kinase-II
CCI	controlled cortical impact
CNS	central nervous system
CSF	cerebrospinal fluid
CT	computed tomography
DAI	diffuse axonal injury
FPI	fluid percussion injury
FRET	Förster resonance energy transfer
GCS	Glasgow Coma Scale
GFAP	glial fibrillary acidic protein
GLI	glibenclamide
GOS	Glasgow Outcome Scale
IMPACT	International Mission for Prognosis and Clinical Trial design in TBI
LHI	large hemispheric infarction
MMP-9	matrix metalloproteinase-9
MRI	magnetic resonance imaging
NFAT	nuclear factor of activated T-cells
NO	nitric oxide
NOS2	nitric oxide synthase-2
OBTT	operation brain trauma therapy
PAR1	protease activated receptor 1
SAH	subarachnoid hemorrhage
SCI	spinal cord injury
Sp1	specificity protein 1
SUR1	Sulfonylurea receptor 1
TBI	traumatic brain injury
TLR4	toll like receptor 4
tPA	tissue plasminogen activator
TRPM4	transient receptor potential melastatin 4
SUR1±TRPM4	SUR1 with or without TRPM4
TUNEL	terminal deoxynucleotidyl transferase dUTP nick end labeling
TXA	tranexamic acid
UCHL1	ubiquitin C-terminal hydrolase-L1
ZO-1	zona occludens 1
